# Intracerebral Haemorrhage in an Adolescent With COVID-19 With Acute Kidney Injury: Is the Virus to Blame?

**DOI:** 10.7759/cureus.14198

**Published:** 2021-03-30

**Authors:** Daisy Khera, Siyaram Didel, Aliza Mittal, Sarbesh Tiwari, Pawan Garg

**Affiliations:** 1 Pediatrics, All India Institute of Medical Sciences, Jodhpur, IND; 2 Diagnostic and Interventional Radiology, All India Institute of Medical Sciences, Jodhpur, IND

**Keywords:** covid 19, intracerebral hemorrhage, acute kidney injury, adolescent

## Abstract

Neurological manifestations in COVID-19 are well described. We describe a 15-year-old girl with acute focal deficit with altered sensorium due to massive right intracerebral hemorrhage following a hypertensive emergency and acute on chronic kidney disease. She was found to be COVID positive by reverse transcription-polymerase chain reaction (RT-PCR). She gradually improved although her neurological deficit in the form of left hemiparesis persisted at discharge. There might be a possible association between intracerebral hemorrhage and COVID-19, although the causation is still not well established.

## Introduction

Patients with severe COVID-19 infection develop pulmonary as well as extrapulmonary manifestations. Complications in patients with severe COVID-19 include hypoxemia, adult respiratory distress syndrome, postviral bacterial superinfection, septic shock, metabolic acidosis, coagulation abnormalities and multiple organ dysfunction [1]. Neurological manifestations in COVID-19 like anosmia, ageusia, encephalopathy, encephalitis, Guillain Barre syndrome and acute cerebrovascular disease are well described [2]. Case reports describing the association between COVID 19 and intracerebral hemorrhage in children/adolescents are scarce.

## Case presentation

A 15 years old previously healthy adolescent girl presented with acute onset focal deficit along with altered sensorium. For these complaints child was taken to a nearby hospital where she developed one episode of generalized tonic-clonic seizure lasting for few minutes which was aborted with medication. She was evaluated with non-contrast computed tomography (NCCT) of brain which was suggestive of large right intracranial (IC) hemorrhage with gangliocapsular bleed with intraventricular extension with midline shift and child was referred to our hospital. There was no history of any trauma, fever, rash, joint pain, decreased urine output, visual blurring, facial puffiness, loose stools, cola colour urine, visible bleeding, icterus or headache.

She had no significant past history. She was a class 12th student and had average school performance and her immunization status was not known. There was no family history of any chronic illness, hypertension, diabetes or chronic kidney disease. Neither the child nor her family members had any typical symptoms of COVID-19 nor had any contact with a known positive.

**Figure 1:**
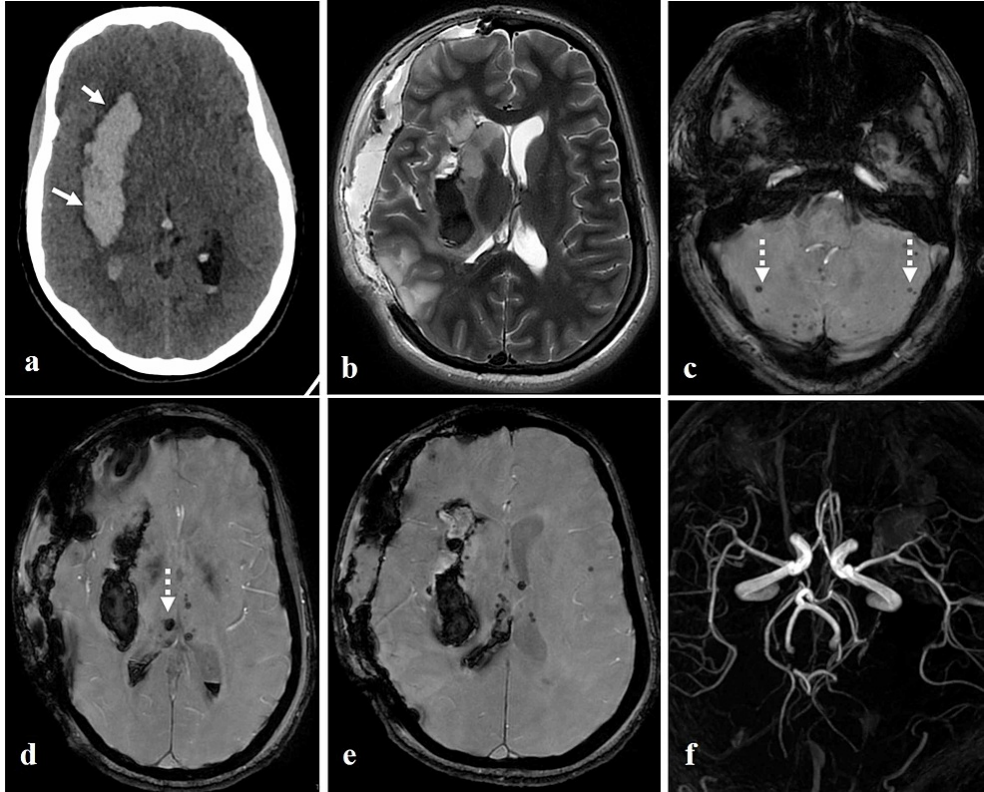
Non-contrast CT brain, post-operative magnetic resonance imaging (MRI) T2W image and MR angiography of brain. The axial non-contrast CT image (a) shows acute intraparenchymal hemorrhage at right gangliocapsular region with intraventricular extension (white arrows). The post-operative T2 image (b) shows right-sided fronto-parietal craniotomy with residual hemorrhage at the right basal ganglia. The axial SWI images (c, d, e) show multiple microhaemorrhage at bilateral cerebellar hemispheres, bilateral thalami, pons and left insular cortex (dashed arrows). The time of flight (TOF) MR angiogram (f) of cerebral vessels is normal.

**Figure 2:**
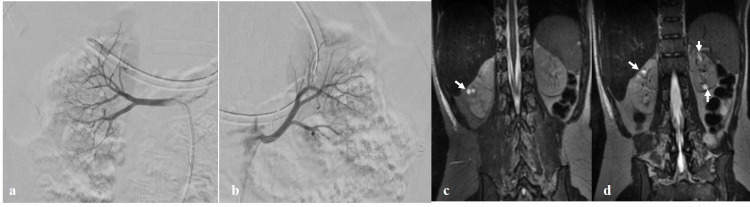
Catheter angiogram of bilateral renal arteries and MRI abdomen. The diagnostic catheter angiogram of bilateral renal arteries (right renal artery (a) and left renal artery (b)) does not reveal any obvious narrowing of renal arteries or their segmental branches. Coronal T2 fat-saturated images of abdomen (c, d) show few cysts within both the kidneys (white arrows). The left kidney is relatively smaller in size (left kidney: 7 cm and right kidney: 8.3 cm).

On receiving in our emergency, she was in hypertensive emergency (blood pressure 160/101 mm of Hg) with altered sensorium and other vitals were pulse rate 90/ min, respiratory rate 20/min, oxygen saturation 100% on room air. Her Glasgow coma scale (GCS) was 11/15 (E3V3M5). Her anthropometry and general physical examination were normal. She had normal cranial nerve examination. On motor examination, she had hypotonia and left-sided hemiplegia (power of 1/5 in left upper and lower limb). Rest of her systemic examination including chest was unremarkable. Her fundus examination was suggestive of grade IV papilledema without any evidence of hypertensive retinopathy. She had deranged kidney function tests at admission. She was noted to have proteinuria (520 mg/24 hrs) but no haematuria or active urinary sediments. Hence initial diagnosis of acute kidney injury (AKI) with hypertensive encephalopathy with right intracerebral haemorrhage was made and treatment started. She was started on medical measures for intracranial hypertension and antihypertensive treatment (labetalol infusion) at admission and was shifted immediately to paediatric intensive care unit for further management. She had gradual worsening of sensorium (GCS became 7) and therefore, was intubated and taken up for surgical intervention in the form of decompressive craniectomy with external drainage. On routine pre-operative testing prior to surgery she was found to be COVID-19 positive by reverse transcription- polymerase chain reaction (RT-PCR). Her chest X-ray at admission was normal. Post operatively her GCS remained less than 8 and hence she was planned for tracheostomy. Her investigations are detailed in Table 1. Her NCCT brain, post-operative magnetic resonance imaging (MRI) T2W image and angiography of brain is shown in Figure 1. Her MRI abdomen and diagnostic catheter angiogram of bilateral renal arteries were normal as is shown in Figure 2. Her kidney function tests worsened during the initial days of hospital stay (maximum creatinine of 2.36 mg/dl) and gradually improved with conservative management (serum creatinine 1.08 mg/dl). Her hypertension was controlled on multiple antihypertensive drugs and proteinuria had resolved at discharge. She also developed polymicrobial sepsis during hospital stay which was treated with appropriate antibiotics and gradually child improved. At the time of discharge, her sensorium is normal but her hemiparesis is persisting. A final diagnosis of hypertensive emergency with right intracerebral haemorrhage with COVID-19 with acute on chronic kidney disease was made.

**Table 1:** Hematological, biochemical and radiological investigations Hb, haemoglobin; TLC, total leucocyte count; N/L, neutrophil/lymphocyte count; hsCRP, high sensitivity C-reactive protein; ESR, erythrocyte sedimentation rate; SGPT, serum glutamic pyruvic transaminase; ALP, alkaline phosphatase; PTH, parathyroid hormone; PT/INR, prothrombin time/international normalized ratio; APTT, activated partial thromboplastin time; ANA, antinuclear antibody; LDH, lactate dehydrogenase; RT-PCR, reverse transcription-polymerase chain reaction; ACE: angiotensin-converting enzyme.

Investigations (normal range)	Day 1	Day 6	Day 42
Hb (11.5-14 g/dL)	11.2	8.3	7.4
TLC (4,000-11,000/mm^3^)	13,370	10,280	3,830
N (22-55%)/L(15-45%)	92.7/4.7	75/11.5	58.8/23.0
Platelet (1.5-4.5 lakhs)	216,000	549,000	285,000
Serum urea (5-18 mg/dL)	32	111	62
Serum creatinine (0.2-0.6 mg/dL)	1.27	2.36	1.08
hs CRP (<1 mg/dL)	5.72	135.74	30
Procalcitonin (<0.05 ng/ml)	1.0	0.11	0.66
ESR (0-10 mm/hour)	5	NA	43
SGPT (9-24 U/L)	08	58.3	76.1
ALP (150-420 U/L)	95	67	94
Calcium (9-11 mg/dL)	8.58	10.4	-
Phosphorus (4-6.5 mg/dL)	4.26	5.24	-
PTH (18.5-88 pg/ml)	77.4	-	-
PT (sec)/INR	12.7/1.09	17.6/1.33	-
APTT (sec)	20.7	36.5	-
D-dimer (<0.5 µg/ml)	-	8.19	8
Fibrinogen (180-350 mg/dL)	-	400	-
Ferritin (10-291 ng/ml)	-	177.8	859.3
Interleukin-6 (<4.5 pg/ml)	-	164.2	-
ANA (<1.5-negative)	Negative	-	-
p-ANCA/c-ANCA	Negative (Titre 1:20)		
LDH (110-295 U/L)	242	403	-
Nasopharyngeal swab RT-PCR for COVID-19	Positive		
Renal doppler	Normal		
Ultrasonography of kidneys and urinary bladder	Bilateral raised renal cortical echotexture with lost cortico-medullary differentiation		
2D echocardiography	Trivial tricuspid regurgitation, concentric left ventricular hypertrophy, normal left ventricular function, no evidence of coarctation of aorta.		
24 hours urine protein	520 mg/24 hrs		
Captopril enhanced DTPA scan	High possibility of renovascular hypertension involving bilateral kidneys with decline of 12.3% and 13.2% of baseline GFR on right and left side respectively post ACE inhibitor.		
Digital subtraction angiography of bilateral renal arteries	Normal		
Serum renin (2.8-39.9 µIU/ml)	16.86		
Serum aldosterone (1.76-23.2 ng/dL)	22.9		

## Discussion

SARS-CoV-2 might display viral tropism and directly affect the kidney. Endothelial dysfunction, coagulopathy and complement activation are likely important mechanisms for AKI in a subset of patients with COVID-19 [3]. In 52 hospitalized children with COVID-19, Stewart et al have reported a high incidence of renal dysfunction (46%) and AKI (29%) [4]. It is well known that SARS-CoV-2 virus uses angiotensin-converting enzyme (ACE) II receptor for cell entry. ACE II is highly expressed in lung alveolar type 2 cells and epithelial cells of gastrointestinal system [5]. They are also expressed in cerebrovascular endothelial cells [6]. Thus, it could be hypothesized that brain ACE II could be involved in COVID-19 infection and its dysfunction leads to disruption of autoregulation leading to intracranial hemorrhage. It has been reported that SARS-CoV-2 patients who have stroke have higher mortality rates [7].

It is clear that extra pulmonary organs are involved in COVID-19 and kidneys are among the most frequently affected. Proteinuria is known to occur in those affected by COVID-19 and it resolves in about two to three weeks in about 70% patients [8]. Our patient was documented to have a sub-nephrotic range proteinuria which could be due to hypertension per se or due to COVID-19 itself. While intracranial hemorrhage may be a complication of hypertensive crises in acute on chronic kidney disease, it is usually seen in those with progressively declining glomerular filtration rates and increasing proteinuria. These may be compounded by the use of antiplatelet or anticoagulant agents [9]. In a previously normal child with no other co-morbidity such a presentation for previously undiagnosed hypertension is uncommon. It is highly unlikely that hypertension would be the cause of such massive intracranial hemorrhage (ICH) in our patient that would require neurosurgical intervention. Was COVID-19 related to the intracranial bleed or was it just co-incidental is an unresolved question. There was no hematological abnormality such as thrombocytopenia or bleeding diathesis identified in our case, nor did she develop cytokine storm.

Sharifi-Razavi et al [10] has reported a 74-year-old man who had fever and cough for three days and was brought unconscious to the hospital and was found to have massive right intracranial hemorrhage and was COVID positive. Aggarwal et al [11] did a retrospective observational study evaluating all stroke cases admitted in their centre for one month (22 March to 21 April 2020) and found that two patients with ICH were SARS-CoV-2 positive and they had no or mild respiratory symptoms and had a higher occurrence of renal dysfunction. They also concluded that there could be a possible association between ICH and SARS-CoV-2 infections. There are many case reports of intracerebral hemorrhage in adults with COVID 19 but reports is children are lacking. Basirjafari et al reported subarachnoid hemorrhage (SAH) as a severe neurological manifestation associated with pediatric COVID‐19 in a 9-year-old child [12].

## Conclusions

The exact pathogenesis of ICH due to SARS-CoV-2 is still not known and the disease has varied manifestations. The potential possibilities leading to ICH in COVID 19 include an infective arteriopathy, viral infection-induced platelet dysfunction or thrombocytopenia, activation of pro-inflammatory cascade leading to cytokine storm or severe viral infection leading to consumption coagulopathy and multi-organ dysfunction. We would like to suggest an association between ICH and SARS-CoV-2 infection. However, larger observational studies are needed to establish the causation.
